# The Role of Cortico-Thalamo-Cortical Circuits in Language: Recurrent Circuits Revisited

**DOI:** 10.1007/s11065-019-09421-8

**Published:** 2019-11-22

**Authors:** Bruce Crosson

**Affiliations:** 1 Department of Veteran Affairs Rehabilitation Research and Development, Center for Visual and Neurocognitive Rehabilitation, Atlanta VA Medical Center – 151R, 1670 Clairmont Rd, Decatur, GA 30033 USA; 2 Department of Neurology, Emory University, 12 Executive Park Drive, Atlanta, GA 30329 USA

**Keywords:** 4–6 thalamus, Language, Aphasia, Semantics, Lexical

## Abstract

Based on a review of recent literature, a recurrent circuit model describes how cortico-thalamo-cortical and cortico-cortical circuitry supports word retrieval, auditory-verbal comprehension, and other language functions. Supporting data include cellular and layer-specific cortico-thalamic, thalamo-cortical, and cortico-cortical neuroanatomy and electrophysiology. The model posits that during word retrieval, higher order cortico-thalamo-cortical relays maintain stable representations of semantic information in feedforward processes at the semantic-lexical interface. These stable semantic representations are compared to emerging lexical solutions to represent the semantic construct to determine how well constructs are associated with each other. The resultant error signal allows cortico-cortical sculpting of activity between the semantic and lexical mechanisms until there is a good match between these two levels, at which time the lexical solution will be passed along to the cortical processor necessary for the next stage of word retrieval. Evidence is cited that high gamma activity is the neural signature for processing in the cortico-thalamo-cortical and cortico-cortical circuitry. Methods for testing hypotheses generated from this recurrent circuit model are discussed. Mathematical modeling may be a useful tool in exploring underlying properties of these circuits.

## Statement of the Problem

Lesions in the left ventral anterior thalamus, which generally are ischemic, almost always result in aphasia acutely characterized by semantic paraphasias often deteriorating into semantic jargon with intact repetition and variability of other symptoms. Left pulvinar lesions, which generally are hemorrhagic, result in semantic paraphasias, fluent output, intact repetition, and comprehension less impaired than the pattern of output would suggest for cortical lesions (Crosson, 1992). As a rule, these aphasias abate rapidly, though incompletely. Symptoms appear to be lexical-semantic in origin (Crosson, 1999; Raymer, Moberg, Crosson, Nadeau, & Rothi, 1997), and the relatively intact repetition resembles transcortical aphasias. More broadly, almost every left middle cerebral artery infarct causing aphasia affects cortico-thalamic connectivity (i.e., cortex, thalamus, and/or connections between them). Hence, understanding the role of the thalamus in language has important implications regarding neural substrates of language.

Six years ago, a reconsideration of thalamic mechanisms in language was published based on advances regarding thalamic mechanisms prior to 2012 (Crosson, 2013). That treatise highlighted four inter-related thalamic mechanisms important for language: (1) cortico-thalamo-cortical circuitry capable of passing information from one cortical area to another via the thalamus, (2) cortico-thalamic circuitry capable of changing the mode of information transfer for thalamo-cortical relay neurons, (3) cortico-striato-pallido-thalamo-cortical circuitry that enhances the efficiency of selecting actions for execution, and (4) cortico-thalamic feedback mechanisms capable of sharpening the focus on specific information necessary for cognitive processes.

However, one unresolved conundrum arising from these conclusions is what purpose cortico-thalamo-cortical information transfer serves when direct cortico-cortical circuitry exists (e.g., see Theyel, Llano, & Sherman, 2010). In other words, it would seem that direct cortico-cortical connections would provide for more efficient transfer of information between cortical areas to support the computational machinery for language. Between 2013 and the current time, the literature has continued to advance at a rapid rate, yielding important information relevant to the resolution of this conundrum. Hence, the current discussion focuses on cortico-thalamo-cortical information transfer. A good starting point is the concept of recurrent circuits.

### Recurrent Circuits

The work of Hebb contained the seeds of a conceptual framework useful in addressing the cortico-thalamo-cortical conundrum. Hebb (1949) proposed the idea of reverberating circuits, which consisted of pathways between multiple neural units in which a pattern of activity conveying information can travel from one unit to the next, eventually returning to the unit from which the activity pattern originated. Hebb conceptualized reverberating circuits as mechanisms to keep information contained in neural firing patterns active to facilitate moving from one step in neural processing to the next. In other words, as a percept or idea unfolds across time, reverberating circuits are a way of bridging time that allows the brain’s computational machinery to translate information from one step of perception into a different code appropriate to the next step. While this idea had merit, it was too simplistic to use in current models for language or other cognitive functions. A central problem is that the reverberation was conceived as unchanging, whereas a dynamic process would be more consistent with our current understanding of the nervous system. A second drawback was lack of a mechanism for terminating reverberation when it was no longer needed because processing had evolved or attention had turned elsewhere.

Hence, the concept of reverberating circuits eventually gave way to the more dynamic construct of recurrent circuits. Wang (2008) provided one good review of how recurrent circuits handle evolving “reverberation” and termination of such activity. The model developed in this review belongs to the recurrent class of neural-circuit models. A particularly useful and common characteristic of recurrent circuits is the dynamic morphing of informational activity in the circuit through feedback processes. We further develop this theme in the model described below, which endeavors to describe the differing roles of cortico-cortical and cortico-thalamo-cortical circuits in language processing.

A foundational principle for this theoretical framework is the concept of a higher-order thalamic relay. The work of Sherman and colleagues (e.g., Llano & Sherman, 2008, 2009; Sherman & Guillery, 2006; Theyel et al., 2010; Usrey & Sherman, 2019) has emphasized that terminal processes of layer 5 cortico-thalamic connections have much in common with fibers passing information from the periphery to the cortex via the thalamus. Specifically, through their firing patterns these cortico-thalamic terminals pass information to thalamic relay neurons that, in turn, convey that information to the next cortical region in a processing chain. In the following sections, we cite evidence that recurrent activity resulting from the interaction of cortico-cortical and cortico-thalamo-cortical circuitry allows for the sculpting of neural activity necessary for high-definition cognitive processing.

## The Cortex and the Thalamus in Information Processing

Language deficits in thalamic aphasia suggest that the thalamus has significant impact on lexical-semantic processes during word selection (Raymer et al., 1997). Hence, since our main focus is what higher-order (cortico-thalamo-cortical) relays contribute to lexical-semantic selection, we begin by discussing structural and physiological characteristics of cortico-thalamo-cortical relays. Subsequently, we cover unique attributes of cortico-cortical connectivity relative to cortico-thalamo-cortical circuitry. Then, we discuss evidence regarding how cortico-thalamo-cortical circuitry interacts with cortico-cortical processing. This latter topic involves discussion of the cortical rhythms indicative of information processing states as well as evidence that the thalamus influences and is influenced by these rhythms. Subsequently, we discuss cortical regulation of thalamic mechanisms. Once we have these building blocks in place, we integrate them into an updated recurrent circuit model integrating both cortico-thalamo-cortical and cortico-cortical mechanisms.

### Cortico-Thalamo-Cortical Relays

In this section, we cover properties of cortico-thalamo-cortical relays. As Sherman and Guillery (2006) stated, a growing literature indicates that thalamic nuclei not only act as first-order gated relays to the brain from the periphery, but also act as a *higher-order gated* relays from one cortical area to another. Specifically, cortico-thalamic fibers originating in layer 5 make similar contacts on thalamic relay neurons as are made by fibers from the optic track, auditory tecto-thalamic projections, or somatosensory lemniscal pathways on their respective thalamic relay neurons. Large terminal processes synapse on the soma and proximal dendrites of thalamic relay neurons making contact exclusively with ionotropic receptors, putting them in position to have a strong and immediate influence on relay neuron firing. Hence, layer 5 cortico-thalamic connections are thought to be drivers of thalamic relay neuron firing patterns. In contrast, layer 6 cortico-thalamic fibers make contact on proximal and distal dendrites with smaller synaptic processes through both ionotropic and metabotropic receptors. These fibers also give off collaterals to the thalamic reticular nucleus as they pass through it. The thalamic reticular nucleus is a thin shell of GABAergic neurons surrounding the thalamus, projecting topographically to the same thalamic nucleus as the layer 6 cortico-thalamic fibers passing through it. In contrast to the layer 5 fibers, layer 6 fibers are thought to modulate the state of thalamic relay neurons. Finally, thalamo-cortical relay neuron projections can terminate in layers 2, 3, or 4, with sparser projections to layers 1 and 6 (Usrey & Sherman, 2019).

Regarding states affected by layer 6 cortico-thalamic projections, we have known for some time that thalamic relay neurons reside in one of two states related to their resting membrane potential (McCormick & Feeser, 1990). In a relatively polarized state, the relay neuron’s baseline firing rate is relatively low, as would be expected, and the neuron fires in high-frequency bursts, with the correspondence between driving inputs and the relay neuron’s output being non-linear. In a depolarized state, relay neurons fire at a higher baseline rate, and there is a linear relationship between input to the relay neuron and its output firing pattern (Sherman & Guillery, 2006). Sherman and Guillery (2006) provided detailed analysis of the properties of these two (nonlinear and linear) information transmission modes. For our purposes, it suffices to recapitulate their conclusions: The linear transmission mode is best suited for high-fidelity transfer of information through thalamic relay neurons and is useful when detailed information processing is necessary. In other words, this transmission mode represents an attentive state with respect to information that is transferred, which allows for detailed processing of that information. On the other hand, nonlinear transmission is best suited for detecting stimuli, but cannot support high-fidelity processing of them. Hence, it represents an inattentive state with respect to the information being transferred, but one in which stimuli can be detected and capture attention in a bottom-up manner, facilitating a switch to the attentive state as necessary.

Although these principles of information transmission were based on work done in first-order sensory relays, Sherman and Guillery (2006) advocated that they can be extrapolated to higher-order (cortico-thalamo-cortical) relays. Further, as Crosson (2013) noted, Sherman and colleagues provided evidence that mechanisms for higher-order information transmission exist. For example, using a mouse model, Llano and Sherman (2008) studied the relationship between primary and secondary auditory cortices and the related dorsal and ventral medial geniculate body (MGB), which are auditory thalamic regions. Tracers injected into primary auditory cortex label cortico-thalamic drivers almost exclusively located in the dorsal MGB. Tracers injected into the dorsal MGB label connections to layers 1, 4, and 6 of secondary auditory cortices. This circuitry provides the mechanism for a higher-order cortico-thalamo-cortical relay from primary to secondary auditory cortices.

Theyel et al. (2010) further examined functional properties of a higher-order cortico-thalamo-cortical circuit in mice. Using flavoprotein autofluorescence in vitro, they showed that stimulating primary somatosensory cortex innervated by barrel fields activates the somatosensory thalamus (posteromedial nucleus), which in turn activates secondary somatosensory cortex. They ruled out the possibility that the activation of secondary somatosensory cortex was caused by direct cortico-cortical connections between primary and secondary cortices by cutting those fibers and observing that primary somatosensory stimulation still activated the corresponding thalamic nucleus and secondary somatosensory cortex. Further, they demonstrated that when inputs to the somatosensory thalamus were blocked with an antagonist to glutamate, stimulating primary somatosensory cortex no longer activated secondary somatosensory cortex, and when the glutamate antagonist was washed out of the thalamus, the ability to activate secondary somatosensory cortex with primary somatosensory cortex stimulation returned. Hence, these observations suggest that it is possible to transmit information from one area of cortex to another via a thalamic relay and that these connections are an example of higher-order thalamic relays as discussed by Sherman and Guillery (2006).

Some other properties of cortico-thalamic neurons are worth mentioning: (1) These pyramidal neurons are more prominent in cortical layer 5b than in layer 5a (Kawaguchi, 2017; Usrey & Sherman, 2019). Morphologically, the basal dendrites from layer 5b cortico-thalamic neurons populate layer 5b more heavily than layer 5a. The apical dendrites in rat frontal cortex branch as they ascend to layer 1, with relatively prolific branches reaching that location (Kawaguchi, 2017). Dendritic branching in layers 2/3 exists but is relatively sparse. Thalamo-cortical projections may contact dendrites of frontal cortico-thalamic neurons at any layer above and including layers 5a and 5b, but the pattern of dendritic branching and, therefore, contact may vary between cortico-thalamic neurons in layers 5a and 5b (Kawaguchi, 2017; Morishima, Morita, Kubota, & Kawaguchi, 2011). On the other hand, the layers of prominent thalamo-cortical contact in primary visual cortex is dependent on whether thalamo-cortical neurons belong to the magnocellular, parvocellular, or koniocellular streams. These streams have implications for both the speed at which information is processed and the level of visuospatial resolution (Usrey & Sherman, 2019). Since, the layer of thalamo-cortical contact may vary from one type of cortex to the next and not all areas of cortex have been mapped (especially in humans), caution should be exercised in generalizing the details across cortices and/or species. For current purposes, the main point is that thalamo-cortical contact makes information conveyed by the thalamic relay neurons available to cortical computational machinery, a concept that will be further addressed in succeeding sections of this review. (2) Cortico-thalamic neurons also send fibers to the striatum unilaterally, various sites in the brainstem, and in some cases the spinal cord and other cortical areas unilaterally (Kawaguchi, 2017; Usrey & Sherman, 2019). Usrey and Sherman (2019) have suggested that the subcortical connections serve to provide efference copies to brainstem locations. What is different about the cortico-thalamic connections, however, is that they provide input to thalamic nuclei for higher-order (cortico-thalamo-cortical) thalamic relays. (3) Layer 5 cortico-thalamic neurons communicate with each other in simple loops; evidence indicates local axonal branching for these cells occurs mainly in layer 5 (Larsen & Callaway, 2006). Excitatory postsynaptic currents from these connections show short-term (~100 ms) facilitation, indicating that repeated stimulation is more likely to fire the neuron than the first stimulation. Further, the firing of cortico-thalamic neurons is relatively slow in changing the firing patterns of other cortico-thalamic neurons (Kawaguchi, 2017; Morishima et al., 2011). This latter property indicates that cortico-thalamic circuitry is tuned to *repeat and perpetuate firing patterns*, which will be important as we develop the role of cortico-thalamo-cortical processing in recurrent circuits. Specifically, we propose that this pattern is particularly important in transmitting from lower- to higher-order cortices a relatively stable representation of the informational state of the lower-order cortex during perceptual processes. This representational pattern of activity, then, can be compared with the emerging informational state of the higher-order cortex to determine how closely the two informational patterns are associated with each other, which also generates error signals (Bastos et al., 2015) highlighting the discrepancies between the lower- and higher-order informational patterns. The purpose of these error signals will be discussed below.

**Fig. 1:**
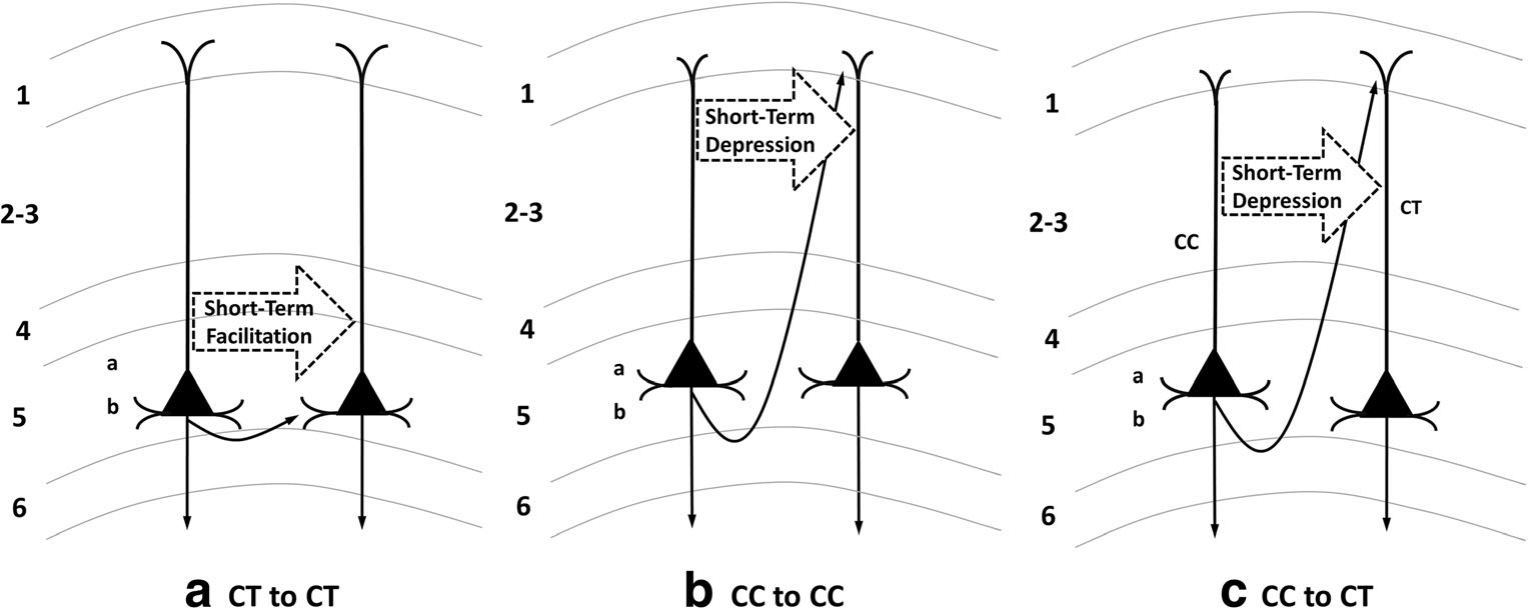
*Connectivity between Layer 5 Pyramidal Neurons*. **a** Cortico-thalamic to cortico-thalamic (CT to CT) cell connectivity probably occurs within layer 5 and causes short-term facilitation of firing between cells. **b** Cortico-cortical to cortico-cortical (CC to CC) connectivity probably occurs in superficial layers and causes short-term depression between cells. **c** Cortico-cortical to cortico-thalamic (CC to CT) connectivity probably occurs in the superficial layers and causes short-term depression of cortico-thalamic cell firing. Cortico-thalamic cells do not influence cortico-cortical cells

Figure 1a shows a likely scenario for cortico-thalamic to cortico-thalamic pyramidal cell contact. While there are some differences in the termination patterns of apical dendrites between mouse somatosensory cortex (Larsen & Callaway, 2006) and rat frontal cortex (Kawaguchi, 2017), for the sake of illustration, we assume that apical dendrites for the canonical cortico-thalamic neuron terminates in layer 1. Finally, it is rare for cortico-thalamic neurons to influence firing patterns in cortico-cortical pyramidal cells (Kawaguchi, 2017). Again, caution is indicated in generalizing these details too literally across cortices and species, but the main point is that these neurons do contact and influence one another in a fashion that tends to perpetuate firing patterns.

### Cortico-Cortical Connectivity

At this point, we reframe the conundrum of the need for cortico-thalamo-cortical transmission of information when direct cortico-cortical fibers exist. Specifically, a better question is how cortico-cortical and cortico-thalamo-cortical processes complement each other in computational systems underlying language processing. To address this question, knowledge of cortico-cortical connectivity is essential. We describe information relevant to the current model and refer readers interested in greater detail to the cited references. Two types of connectivity are relevant: (1) the connectivity between layers within a cortical area that defines cortical columns (intra-area connectivity), and (2) the connectivity between cortical areas that defines cortico-cortical connectivity (inter-area connectivity).

#### Intra-Area Connectivity

In contrast to neurons in layer 5b predominantly giving rise to cortico-thalamic connectivity, layer 5a pyramidal neurons primarily support cortico-cortical connectivity (Kawaguchi, 2017; Usrey & Sherman, 2019). Differing characteristics and processes for cortico-cortical than cortico-thalamic pyramidal cells suggests that the two neuron types make different, but related, contributions to information processing. Some of these differences are as follows: (1) Layer 5 cortico-cortical cells tend to show less basal dendritic branching than cortico-thalamic neurons, and the branching points for these dendrites are more in layer 5a than 5b. The lesser branching is true for apical dendrites as well; i.e., the dendritic processes reaching layer 1 branch less prolifically for cortico-cortical than for cortico-thalamic neurons. Dendritic processes in layers 2/3 are more likely to be located in the upper than the lower parts of layers 2/3, at least for frontal systems (Kawaguchi, 2017). (2) Unlike connections between cortico-thalamic pyramidal cell pairs, local connections between cortico-cortical cell pairs tend to result in a degree of short-term (~100 ms) depression (Kawaguchi, 2017), at least in frontal cortex. Contact between cortico-cortical pyramidal cells may occur in superficial layers where local branching is most prolific, though branching in deeper layers also occurs (Kawaguchi, 2017; Larsen & Callaway, 2006) (Fig. 1b). Hence, assuming generalization to other cortices, while contacts between cortico-thalamic cells tend to promote maintenance of firing patterns, contacts between cortico-cortical pyramidal cells *tend to promote rapidly adapting change in firing patterns*. In other words, cortico-thalamic circuitry is biased toward maintaining information encoding in firing patterns, while cortico-cortical circuitry is biased toward change in information processing (Kawaguchi, 2017). We propose that this property of cortico-cortical cells (at least in layer 5) is useful when neural code from lower-order cortex is translated to neural code in higher-order cortices because the latter process involves massive changes in neural code (e.g., translating lexical to semantic code). In other words, a role of layer 5 cortico-cortical neurons in computational machinery of the cortex may be to promote the substantial changes from input of lower-order cortex that it takes to translate to the associated code in higher-order cortex. (3) Finally, cortico-cortical cells do contact cortico-thalamic cells, likely in superficial layers (Fig. 1c), and the impact on cortico-thalamic cells is that of short-term depression (Kawaguchi, 2017).

**Fig. 2:**
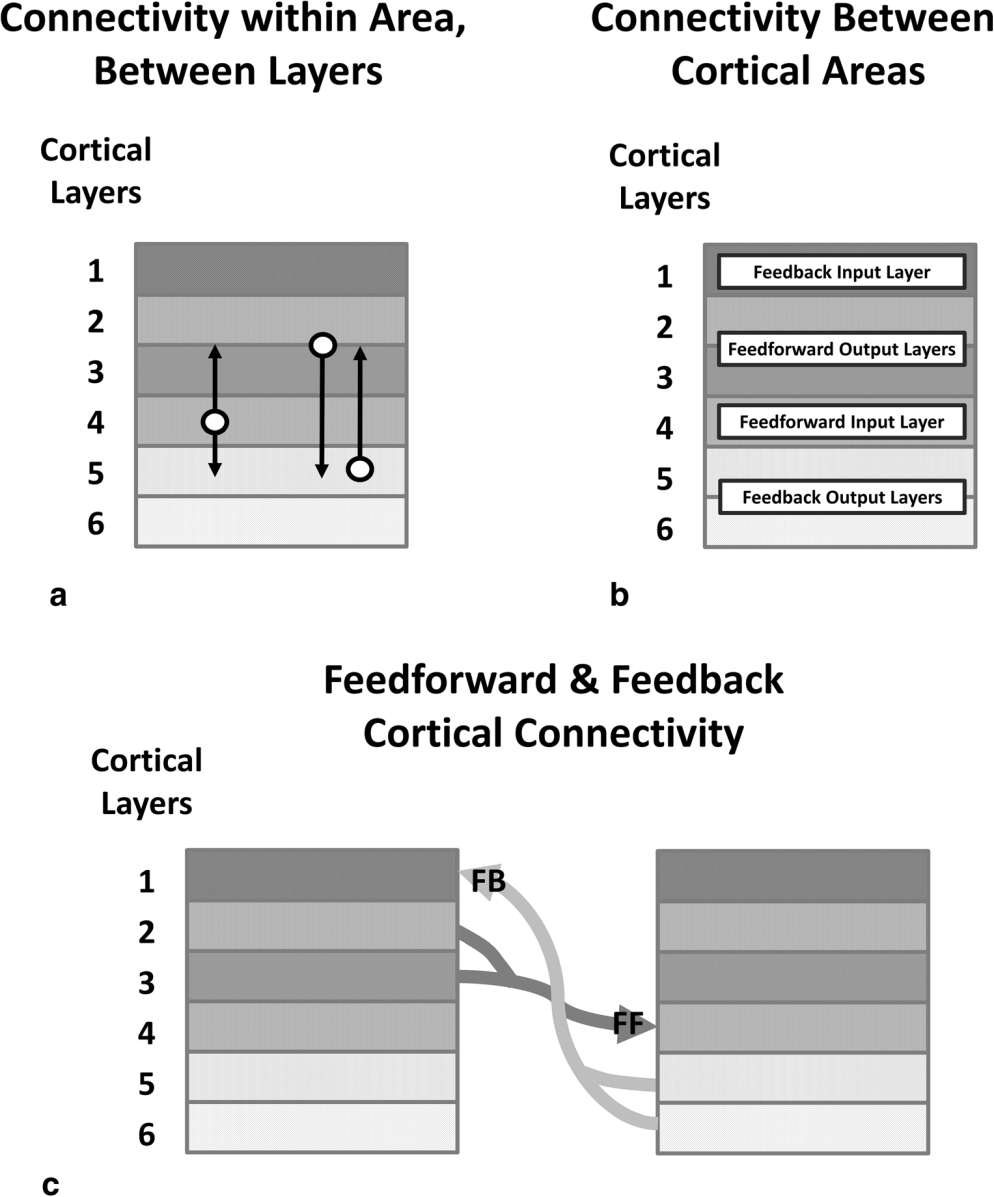
*Between-Layer Cortico-Cortical Connectivity Within and Between Cortical Areas*. **a** Connectivity between cortical layers within a cortical area is reciprocal for layers 2/3 and layer 5. However, layer 4 sends fibers to both layers 2/3 and layer 5, but receives its input from outside its area from a lower-order cortical area and/or from thalamic nuclei. **b** Layer functions are indicated, with layer 1 receiving feedback from higher-order cortex, layers 2/3 sending feedforward output to higher-order cortex, layer 4 receiving feedforward input from lower-order cortex, and layers 5/6 sending feedback to lower-order cortex. **c** Or, pictured differently, layers 2/3 of lower-order cortex sends feedforward output to layer 4 of higher-order cortex, and layers 5/6 in higher-order cortex send feedback to layer 1 of lower-order cortex

Relevant to the cortical computational machinery necessary to translate lower-order input to higher-order neural code are the patterns of connectivity between cortical layers (Fig. 2a). For example, cortical layer 4 receives input from lower-order cortices, or in the case of primary sensory cortices, from the thalamus. Cortical layer 4 sends outputs to cortical layers 5 and 2/3, but does not receive input from those cortices. Hence, layer 4 can be considered an input layer (Harris & Shepherd, 2015). Layers 2/3 and layer 5 have bidirectional connections with each other (Harris & Shepherd, 2015) and, hence, appear to be involved in computational processes based upon layer 4 input.

#### Inter-area connectivity (Fig. 2c)

Since output to higher-order cortices emerges from layer 2/3, it can be considered an output layer, though cortico-cortical connectivity can emerge from layer 6 as well (Kawaguchi, 2017; Pandya & Yeterian, 1985). The layers in which cortico-cortical fibers terminate can vary, depending upon hierarchical relationships, and how the outputs are generated is incompletely understood (Kawaguchi, 2017). For posterior cortices, then, feedforward connections from lower- to higher-order sensory cortices originate in layers 2/3 of the lower-order areas and terminate in layer 4 of the higher-order area. However, feedback connections from higher- to lower-order sensory cortices originate in layers 5 and 6 of the higher-order cortex and terminate in layer 1 of the lower-order cortex (Kawaguchi, 2017; Pandya & Yeterian, 1985). Thus, different cortical layers seem to specialize in feedforward output (layers 2/3), feedforward input (layer 4), feedback output (layers 5 and 6), and feedback input (layer 1) (Fig. 2b). Models suggest that this architecture allows for iterative processing cycles between lower- and higher-order cortices (Rummelhart, McClelland, & Group, t. P. R, 1986).

### Cortical and Thalamic Rhythms and Information Processing

To understand cortico-thalamic relationships during information processing, we must consider recent studies about cortical rhythms. Some Twentieth Century viewpoints regarding cortico-thalamic relationships emphasized the thalamus as the brain’s (EEG) rhythm generator. Just as the idea of the thalamus as a relay from periphery to cortex is giving way to the inclusion of higher-order cortico-thalamo-cortical relays (Sherman & Guillery, 2006; Usrey & Sherman, 2019), the concept of the thalamus as the brain’s rhythm generator is being replaced by the idea that cortical rhythms are generated by an interaction between cortico-thalamic as well as thalamo-cortical influences (Steriade, 2006). An observation particularly relevant for our model is that thalamo-cortical relay neurons fire in synchrony with cortical gamma rhythms, suggesting a relationship between this cortical rhythm and thalamic firing (Steriade, Contreras, Amzica, & Timofeev, 1996). Such fast cortical rhythms (in the beta and gamma ranges) occur when cortical neurons are in a state of relative depolarization. Further, fast cortical rhythms can show synchrony from one cortical area to the next (Edwards et al., 2010; Steriade, 2006). Recent data suggest specific roles in information processing regarding such fast cortical rhythms.

With respect to language, using electrocorticography, Edwards et al. (2010) showed that language processing was accompanied by large, significant temporary increases in the magnitude (analytic amplitude) of high gamma rhythms (70–160 Hz). Further, the temporal profile of these high gamma rhythms varied dependent on the function of the cortex represented in specific recording channels. Specifically, presentation of the auditory stimulus (noun) for a verb generation task was accompanied by increased amplitude of high gamma rhythms in Wernicke’s area (mid to posterior left superior and middle temporal gyri) as well as by activity in the left precentral gyrus. As processing switched to word retrieval, high gamma activity in Broca’s area (left pars opercularis, pars triangularis) emerged, and closer to actual production of the verb, such activity was seen peri-Rolandic cortices. High gamma activity during word retrieval and production phases of picture naming was generally consistent with the activity shown during verb generation, as well as with known functions of language eloquent cortices. It is further worth noting that the blood oxygenation level dependent (BOLD) signal used to demonstrate brain activity in functional magnetic resonance imaging (fMRI) studies is closely associated with local field potentials in the gamma range in a variety of cortices (premotor, association, visual, auditory) (Brovelli, Lachaux, Kahane, & Boussaoud, 2005; Lachaux et al., 2007; Logothetis, Pauls, Augath, Trinath, & Oeltermann, 2001; Mukamel et al., 2005). Indeed, high gamma activity shown by Edwards et al. (2010) during word retrieval and production is generally consistent with our own fMRI findings for word generation (e.g., Meinzer et al., 2009; Meinzer et al., 2012) and picture naming (Wierenga et al., 2009).

Even beyond language, evidence is mounting that gamma band synchronization is fundamental to cortical computations underlying cognition (Fries, 2009). In studies of working memory in macaques, Miller’s lab has shown that neural spiking indicative of information processing in prefrontal cortex occurs only during brief bursts of gamma band activity (45–120 Hz). These gamma bursts occur both during encoding of information and when the information is retrieved to meet demands of the working memory task (Lundqvist, Herman, Warden, Brincat, & Miller, 2018; Lundqvist et al., 2016). Gamma band power increases with increasing working memory load (Lundqvist et al., 2016). Keeping in mind that increased BOLD fMRI signal represents gamma band activity (Brovelli et al., 2005; Lachaux et al., 2007; Logothetis et al., 2001; Mukamel et al., 2005), human verbal working memory shows an analogous phenomenon. Specifically, in a BOLD activation study of verbal working memory, Moore, Li, Tyner, Hu, and Crosson (2013) showed an area of left dorsolateral prefrontal cortex that was active during both encoding and retrieval of lexical-semantic information, and is accompanied by neostriatal and ventral anterior thalamic activity during encoding.

Beta band activity is negatively correlated with gamma band activity in frontal cortex during working memory. In other words, when power in the gamma band increases, power in the beta band decreases, and vice versa (Lundqvist et al., 2016, 2018). Lundqvist et al. (2018) suggested that the pattern of beta band power during working memory experiments indicates that it plays a control function, “determining when and where sensory information is processed and retained.” (p. 9) Bastos et al. (2015) addressed the relationship between gamma and beta activity in sensory cortices, specifically different levels of visual cortices. These investigators found that gamma band (and theta) activity was related to feedforward (bottom-up) processes and beta band activity was related to feedback (top-down) processes. Hence, as visual processing proceeds through its various stages, both feedforward and feedback processes occur. This finding is relatively consistent with Lundqvist et al. (2018) suggestion that beta activity is associated with top-down control of information processing, though the function of feedback mechanisms in sensory processing might be more to sculpt feedforward processes to sharpen the focus on relevant information as visual processing unfolds (Sillito, Cudeiro, & Jones, 2006). Indeed, Crosson (2013) explored the implication of such feedback mechanisms for language in some detail. As noted above, Bastos et al. (2015) proposed that feedback consists of error signals from the higher-order to the lower-cortex about congruence of the emerging higher-order percept with the lower-order information input. This error signal iteratively sculpts the feedforward information from the lower-order cortex until the lower-order input is in congruent with the evolving higher order percept.

In widely distributed semantic networks, with information arriving at polymodal cortices from a variety of unimodal cortices, the iterative sculpting of information from multiple sources seems to require on the order of hundreds of milliseconds to achieve resonance between input and resolution of the evolving percept in different processing stages (e.g., see Edwards et al., 2010). Raymer et al. (1997) showed that in thalamic aphasias, semantic and visual-semantic errors in word retrieval were particularly likely for words with low frequency of occurrence in one’s native language. Based on these findings, we suggest that less frequently encountered/used lexical-semantic concepts require greater thalamic participation in coordination of input between lexical-semantic nodes to achieve resonance. This concept of resonance is similar to that of attractor states in parallel distributed processing models (Nadeau, 2012; Rummelhart et al., 1986) and with the same concept applied to recurrent neural circuits (Wang, 2008).

In summary, high gamma band activity is associated with information processing in the cortex, including language functions. This observation is consistent with the fact that gamma band activity drives the hemodynamic changes seen in BOLD fMRI. Gamma band activity in the thalamus is associated with gamma band activity in the cortex, suggesting an association between the two phenomena. We now turn to a potential mechanism for regulating this relationship.

### Cortical Regulation of Thalamic Relay Neuron Firing Modes

What keeps cortico-thalamo-cortical relays from continuing firing patterns established during information processing? How do thalamic neurons switch back and forth between high fidelity (linear) and lower fidelity (nonlinear) firing modes? Some regulatory mechanism is needed to allow switching from one concept to another as lexical-semantic processing proceeds. As we noted above, cortical layer 6 input to the thalamus is thought to change the state of target thalamic relay neurons from a low-fidelity to a high-fidelity transfer mode, where output is linearly related to the driving input. In higher order relays, such as those involved in language, this driving input to the thalamic relay cells comes from layer 5 neurons (Sherman & Guillery, 2006). But, how does layer 6 input to thalamic relay nuclei shift it from a low-fidelity to a high-fidelity transfer mode? We had previously suggested that frontal connections excited neurons in the thalamic reticular nucleus (TRN), which in turn inhibited local inhibitory interneurons in the thalamus, thereby allowing the thalamic relay neurons to depolarize to the lower resting membrane potential that provokes a change to the high-fidelity transfer mode (Crosson, 2013; Nadeau & Crosson, 1997).

However, recent literature suggests a different mechanism than previously proposed (Crosson, 2013; Nadeau & Crosson, 1997) by which layer 6 input may change thalamic relay neurons to the high-fidelity firing mode. Crandall, Cruikshank, and Connors (2015) showed that a single stimulus of layer 6 input to thalamic relay nuclei causes a brief excitatory post-synaptic conductance in relay cells followed by a longer and larger inhibitory post-synaptic conductance mediated by inhibitory cells in the TRN. However, with 10 Hz stimulation, excitatory responses are facilitated and inhibitory responses are suppressed. If this kind of input is substantial, then the membrane potential of the relay neuron should depolarize to a lower resting membrane potential, leading to a change to the high-fidelity firing mode.

**Fig. 3:**
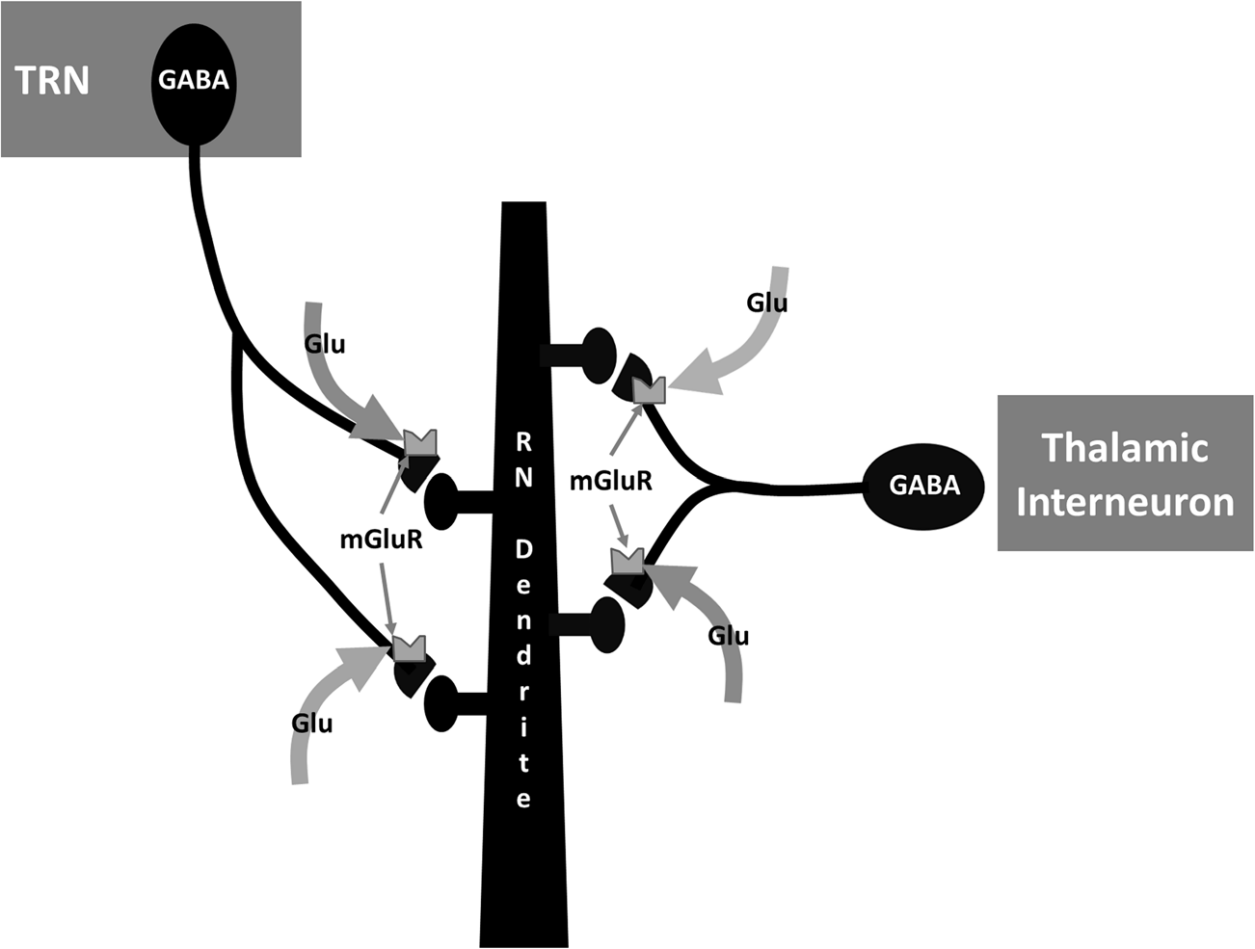
Presynaptic metabotropic glutamate receptors (mGluRs) on terminals from the thalamic reticular nucleus (TRN) or thalamic interneurons synapsing on thalamic relay neuron (RN) dendrites reduce postsynaptic inhibitory conductances when activated. It is hypothesized that when layer 6 corticothalamic connections activate these mGluRs, the depolarization of the RN switches it from a nonlinear (low-fidelity) to a linear (high-fidelity) transfer mode, enabling high-fidelity cortico-thalamo-cortical information transfer. Based on Liu, T., Petrof, I., & Sherman, S. M. (2015). Modulatory effects of activation of metabotropic glutamate receptors on GABAergic circuits in the mouse thalamus. *J Neurophysiol, 113*(7), 2646–2652. 10.1152/jn.01014.2014

Liu, Petrof, and Sherman (2015) described a mechanism that might contribute to the suppression of inhibition (Fig. 3). Activation of presynaptic metabotropic glutamate receptors (groups I and II mGluRs) on terminal processes of TRN or intra-thalamic GABAergic neurons (contrary to what some might expect) leads to reduction of postsynaptic inhibitory conductance on the respective relay cell. Liu et al. suggested that one possible mechanism by which these mGluRs could be activated is spillover of glutamate from nearby synapses, which would be more likely during at a stimulus rate of 10 Hz than during a single input, explaining the findings of Crandall et al. (2015). Fibers from cortical layer 6 are known to give off collaterals to the GABAergic neurons in the TRN as they pass through the latter as well as reaching GABAergic interneurons in their target thalamic nucleus (Sherman & Guillery, 2006). Hence, as glutamatergic activity from layer 6 connections increases and activates MGluRs on GABAergic terminals, inhibitory (GABAergic) influences from TRN and/or GABAergic interneurons should decrease. The resultant release from inhibition would facilitate depolarization of thalamic relay neurons to a lower resting membrane potential, triggering a change to the high-fidelity (linear) mode for information transfer to cortical targets, which as noted above, is tuned for detailed information processing (Sherman & Guillery, 2006). When transfer of information from the relay neuron to its cortical target is no longer necessary, a reduction in layer 6 input would allow inhibitory influences to be re-established, raising the resting membrane potential of the thalamic relay neuron to a more polarized level during which high-fidelity information transfer ceases. The resultant temporary cessation of high-fidelity information transfer via cortico-thalamo-cortical circuitry would allow for new patterns of information transfer to be established when the next cycle of high-fidelity information transfer is initiated via reduction in inhibitory influences (as explained above).

Previously, we demonstrated pathways from both left pars triangularis and pars opercularis (Broca’s area) to the pulvinar and ventral anterior nucleus of the human thalamus (Bohsali et al., 2015), but we did not explore other frontal regions or their thalamic targets. Nonetheless, if human fronto-thalamic projections follow a pattern similar to that of macaques, then every, or nearly every, frontal cortical area will project to the ventral anterior nucleus and the pulvinar (Goldman-Rakic & Porrino, 1985). (When damaged, these nuclei in the left hemisphere consistently lead to thalamic aphasia.) Indeed, some frontal regions may connect with as many as 10 thalamic nuclei (Morel, Liu, Wannier, Jeanmonod, & Rouiller, 2005). Specifically, such connectivity would allow intentional mechanisms in the frontal cortex to selectively engage those neural nets necessary to accomplish the task at hand (Crosson, 2013; Nadeau & Crosson, 1997). However, the work of Crandall et al. (2015) suggests layer 6 input from posterior (sensory) cortices also may reduce the inhibitory influences on thalamic relay neurons, allowing for bottom-up influences as information processing cascades from one stage to the next.

## Recurrent Circuits Revisited

**Fig. 4:**
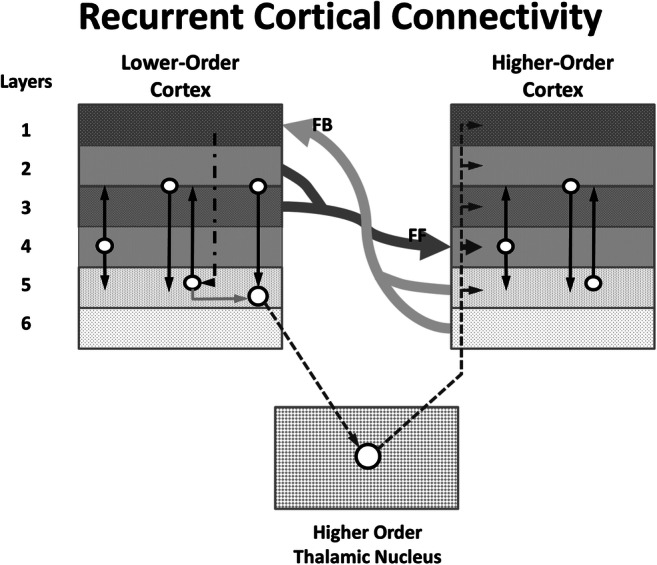
This figure shows the recurrent circuitry between a lower- and a higher-order cortical region that consists of the integrated function of a cortico-cortical circuit between these areas and a cortico-thalamo-cortical circuit between these areas, as well as layer-to-layer circuits within cortical areas. The cortico-cortical circuit feeds forward neural code from lower- to higher-order cortex so that the lower-order code can be transformed into higher-order code. For example, once auditory information has been recognized as a word in cortex that processes lexical information, it can be passed along to cortex that processes semantic information. The layer-to-layer higher-order circuits transform lexical into semantic information to understand the word’s meaning. This transformation from one code to another requires rapid changes in firing patterns between neurons within and between cortical layers. As this transformation is occurring, the cortico-thalamo-cortical circuit maintains a stable representation of the lower-order code that is passed from lower- to higher-order cortices, which allows the higher-order cortex to determine if the emerging higher-order code is usually associated with the lower-order representation. Mismatches between the emerging higher-order code and the lower-order representation results in an error signal that is fed back to the lower-order cortex. This error signal prompts the lower-order cortex to refine the information that is fed forward to the higher-order cortex, beginning a new iteration of processing between the lower- and higher-order cortices. After a few iterations of these cycles, the lower- and higher-order representations reach a resonance indicating successful translation of the lower- to the higher-order representation

Now that the building blocks of our model are established, we can describe how the components work together to process language (Fig. 4). A desirable characteristic of recurrent circuit models (Wang, 2008) is that they allow for dynamic reverberation of information between neural units through which information processing evolves to resolve perceptual and output processes. In our model, we propose (1) distinct roles for cortico-thalamo-cortical vs. cortico-cortical circuits, (2) invoke metabotropic glutamate receptors as the mechanism that modulates firing modes of thalamic relay neurons, and (3) describe dynamic reverberation between cortical lexical and semantic processors that explains word comprehension and word-finding symptoms in thalamic aphasia. Progressing through the steps of word comprehension or word retrieval requires management of a dynamic tension between maintaining the current pattern of activity and provoking the activity pattern necessary for the next stage of information processing, consistent with the properties of a recurrent circuit model.

**Fig. 5:**
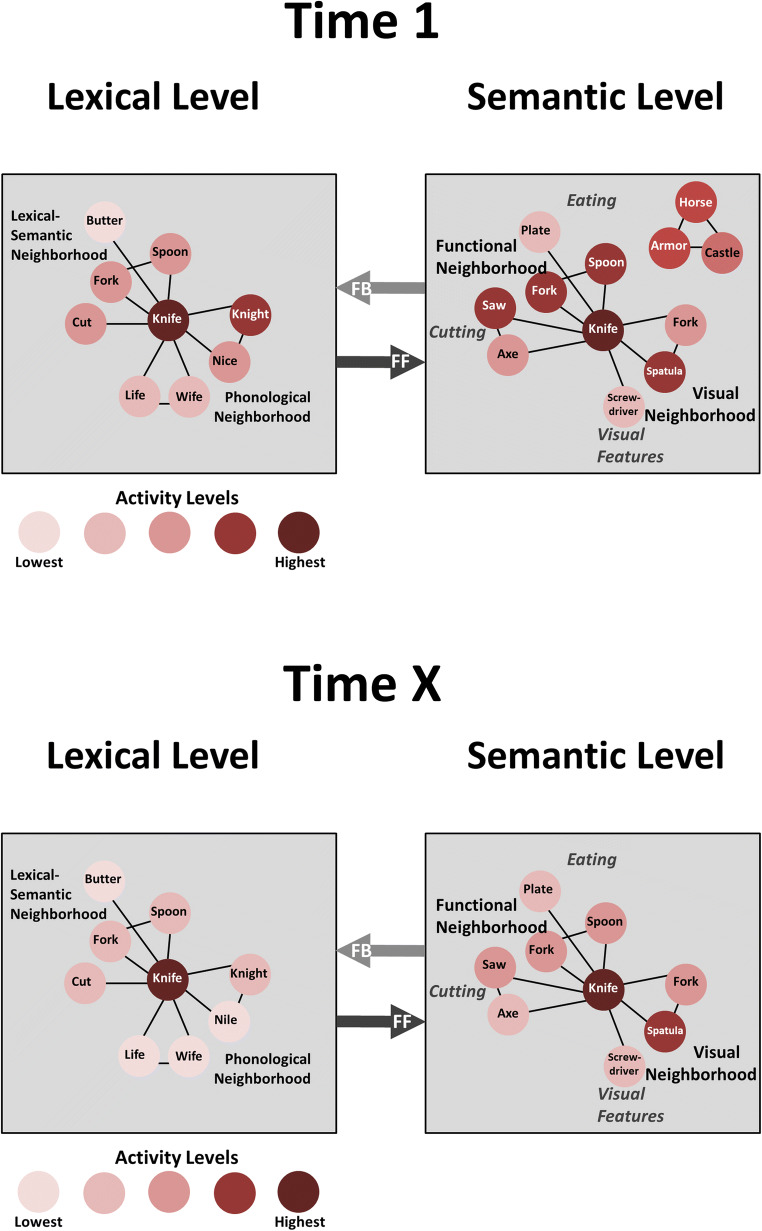
*Auditory-Verbal Stimulus Decoding at the Lexical-Semantic Interface*. At Time 1 (top panel), lexical definition of a heard word, “knife”, is sufficient to begin the process of semantic decoding by passing lexical information on to the semantic level. However, the lexical definition is not at an optimal state because the activity of “knight” is close enough to that of “knife” that there is some probability of confusing the two lexical items. (Note the color scale, with the deepest rust-color representing the highest level of activity.) At Time 1, that potential for confusion also is reflected at the semantic level of processing because the relatively high activation of “knight” at the lexical level has led to a moderately high activation level of items at the semantic level related to “knight”. At Time X (bottom panel), after one or more cycles of feedback followed by feedforward at the lexical-semantic interface, not only has the semantic processing reached a better definition between activation levels of concepts, but as a result of this iterative processing, items at the lexical level also have reached a more nearly optimal definition of activation levels between items

Figures 5 shows our model of how information processing proceeds from one stage to the next. For the purposes of this example, we assume the progression of information through successively higher order cortices in auditory processing, for example, as words in a sentence are successively being decoded. The lower-order cortex (LOC) performs an earlier, less refined stage of information processing and passes the results along to higher-order cortex (HOC), where the ensuing stage of processing takes place. While we propose serial activation of different language processors at different processing stages, several processors could be active simultaneously, depending on processing demands, as suggested by many parallel distributed processing (PDP) paradigms (e.g., Nadeau, 2012).

In this model, the balance between maintaining activity patterns to allow for feedback and translating lower-order information into higher-order constructs at the next processing stage happens at the interface between cortico-cortical and cortico-thalamo-cortical circuits. As indicated above, cortico-cortical circuits are biased toward letting current activity patterns subside (Kawaguchi, 2017), allowing new patterns to emerge. Without some separate mechanism, therefore, information would not remain active to allow for determining the correspondence between lower- and higher-order information signals and generating error feedback to lower-order cortex (as proposed by Bastos et al., 2015). Cortico-thalamo-cortical circuitry, however, is biased toward maintaining activity patterns (Kawaguchi, 2017), and as long as this circuitry is active, ongoing patterns remain active. This functional difference between the two cortex-to-cortex processing circuits, one direct and the other mediated by thalamic nuclei, permits dynamic reverberation as follows.

Based on cortico-cortical input from the LOC, the related pattern of activity at the next processing stage will begin to emerge in the HOC. For example, a lexical form in a LOC processor might act as input to a HOC semantic processor which activates an associated meaning (see Fig. 4 and caption for details). As input from the LOC begins to activate related patterns in the HOC, a stable representation of the state of the LOC is passed to the HOC by cortico-thalamo-cortical transmission. When the emerging construct in the HOC is compared to the informational state of the LOC, an error signal identifying mismatches between the lower- and higher-order activity patterns in HOC is fed back via cortico-cortical connections to the LOC, which, in turn, triggers suppression of residual LOC information not relevant to the emerging HOC construct. A mismatch in the patterns results in a sculpting (or refining) of information in the LOC, in turn resulting in a refinement of the LOC input to the HOC. This proposed process is similar to the error processing between LOC and HOC in the macaque visual system proposed by Bastos et al. (2012, 2015). The cycle of feedforward and feedback between the LOC and the HOC continues iteratively (i.e., dynamically reverberates) until a good coherence between the two patterns occurs, similar to models of parallel distributed processing (Rummelhart et al., 1986). As the coherence is close to resolution, the HOC passes information along to the next stage of information processing. Hence, while this processing model capitalizes on the tendency of cortico-thalamo-cortical processing to maintain patterns of neural activity, that pattern of activity is not just maintained, it is dynamically sculpted to achieve coherence with emerging patterns at the next level of processing. The bias of cortico-thalamo-cortical circuits to maintain firing patterns allows for comparison of LOC output with emerging HOC activity. Error signals generated by this comparison then lead to dynamic sculpting by cortico-cortical circuitry that eventually leads to coherence between LOC and HOC processing. This coherence is based on prior association LOC and HOC states, which must be instantiated through learning processes.

Once coherence between the LOC and HOC is achieved, the next higher level of processing begins to resolve. At this time, the information carried by the activity pattern in the LOC is no longer needed, and the layer 6 input to the thalamic nucleus from the LOC can subside, which reduces the glutamatergic input to pre-synaptic mGluRs on GABAergic terminals that suppress the inhibitory influence of TRN or local GABAergic interneurons. Thus, this reduction of layer 6 glutamatergic input allows for inhibition of the thalamic component of cortico-thalamo-cortical activity, interrupting reverberation in the circuit and making way for the next incoming informational firing pattern. Indeed, when more incoming information to the LOC is anticipated, for example, when more words from an incoming sentence are to follow, frontal input can re-engage thalamic relay neurons in the high-fidelity transfer mode, in readiness to process the next word. The temporary abatement of thalamo-cortical signal when reverberation is halted may be manifested at cortical levels by a change to a lower range of the fast rhythm spectrum, perhaps low gamma or beta. This concept is consistent with the interplay and fast switching between beta and gamma rhythms emphasized by Steriade (2006). It is also consistent with Lundqvist et al. (2018) idea that beta rhythms may be associated with frontal control processes (i.e., the layer 6 input patterns that switch thalamic relays from one transfer mode to the other).

## Applying the Cortical/Thalamic Recurrent Circuit Model to Word Finding

Up to this point, we have focused our recurrent circuit model on how processing evolves from lexical to semantic processing in resolving the meaning of a heard word. While this analysis explains the comprehension problems frequently found in thalamic aphasia, the most severe and persistent symptom in thalamic aphasia is word-finding difficulty. Specifically, patients with thalamic aphasia have lexical-semantic deficits leading to word-finding problems (e.g., written or oral naming responses for pictures or definitions). As a result, they substitute semantically related words for the target word (Raymer et al., 1997), which in the early stages of recovery can lead to semantic jargon in narrative production (Crosson, 1984, 1992; Crosson et al., 1986).

**Fig. 6:**
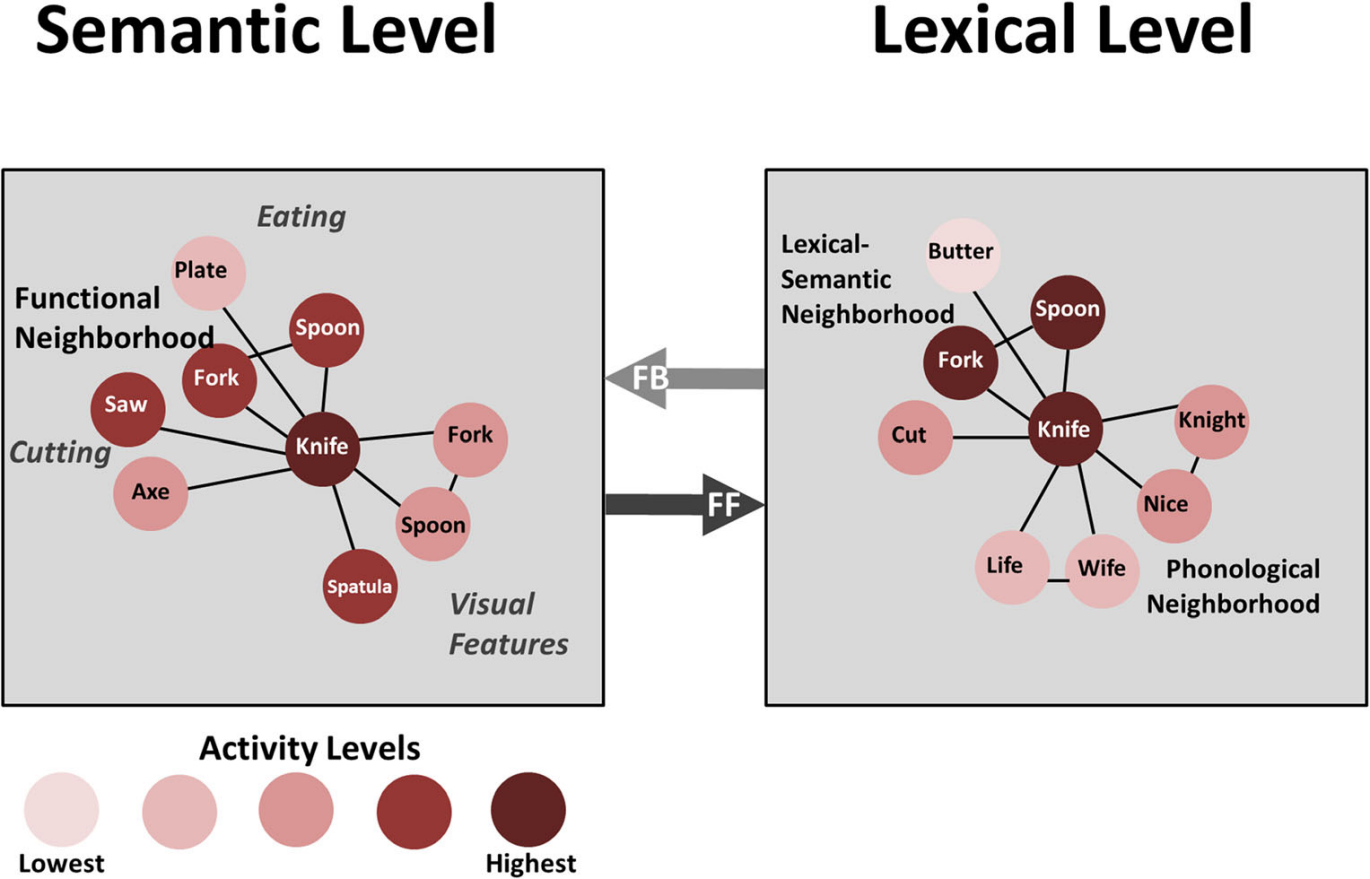
For word-finding, it is necessary to consider how the semantic and lexical processors progress from a concept in semantic cortices to a word-form in lexical cortices. This figure emphasizes both functional and visual associations in semantic cortices. The conceptual representation of “knife” activates “fork” and “spoon” because they have a close function related to that of “knife”, i.e., used in eating. However, “knife”, “fork”, and “spoon” also are objects of similar visual scale, and all have a handle with varying shapes at the end. Hence, because of some visual similarities, additional activation will accrue to “fork” and “spoon”. These different sources of activation for “fork” and “spoon” are additive as they are fed forward to lexical cortices, resulting in nearly equal levels of activation for the words “knife”, “fork”, and “spoon”. When the thalamic component of the recurrent circuitry is eliminated through lesion of the associated left thalamic nucleus, no error signal can be generated, leaving the activation of the three words at nearly equal levels. This condition leads to a high probability of visual-semantic confusion during word retrieval, consistent with the findings of Raymer et al. (1997) for thalamic aphasia

The following discussion depends to some degree on understanding three concepts in PDP models (e.g., Dell, Schwartz, Martin, Saffran, & Gagnon, 1997; Nadeau, 2012; Rummelhart et al., 1986) (See Fig. 6). In different levels of the language domain (e.g., semantic, lexical, or phonemic), representations compete with each other for recognition or expression. For example, in referring to my car, I could choose any of the following: “car”, “automobile”, “Corvette”, or “sports car”. The first PDP concept is “activity level”, which is a representation of the likelihood that one of these items will be selected over the others. The item with the highest activity level is more likely to be selected than the other items. The second PDP concept is that of connection strength. Connection strength between items is a representation of how closely items at some level of processing are associated. For example, at the semantic level of processing, fork and spoon have a higher connection strength with each other than with a pencil because, while all these objects can be manipulated manually, fork and spoon are used for eating and a pencil is not. Fork has a higher connection strength with pencil than mountain because the latter cannot be manipulated manually. Activation level and connection strength can be thought of as derivatives of neural activity and connections. The third PDP concept is that resolution of a stimulus identity or choice of a word to represent a concept is an iterative process between multiple sequential levels of processing that serves to resolve ambiguities in stimulus recognition or choices for expression.

As we turn our discussion from comprehension to expression, we must consider how the problem of word-finding differs from the problem of understanding the meaning of verbal input. In many respects, this dichotomy comes down to understanding the difference between perception of a stimulus vs. planning and executing an action, and is closely related to Heilman, Watson, and Valenstein’s (2012) conceptualization of the dichotomy between attention governing perception vs. intention governing action. Specifically, in resolving the meaning of heard word, the processing is highly constrained by the specific input. While various stages of processing carry along activation of various associations appropriate to the processing level (e.g., lexical associations for lexical processing, semantic associations for semantic processing), the goal of processing is to derive the meaning of the specific stimulus word. In other words, the specific input constrains the eventual semantic resolution of the stimulus. Further, by placing relevant thalamic nuclei in the linear information transfer mode, high-resolution processing of relevant stimuli is afforded.

In contrast, word finding starts off with the intention to express a semantic concept that triggers a lexical search, then the appropriate phonemic sequence and eventually motor execution of a selected word. At the interface between the semantic and lexical systems, where deficits in thalamic aphasia reside (Raymer et al., 1997), there may be several viable lexical choices for the concept a speaker wishes to convey. Consider the example of how to refer to my car, which was just given. The existence of multiple lexical choices, especially when contextual constraints are low, presents a problem for lexical selection because some of the best choices are separated by small activation increments, increasing the difficulty of lexical selection.

During word selection, semantic processing originates in higher order cortex, and triggers sequential activation for lexical, phonemic, and motor planning mechanisms. In other words, processing moves from a higher-order towards primary (motor) cortex, which is the opposite direction from sensory processing, which moves from primary to higher-order cortex (as noted above). Specifically addressing the semantic-lexical interface relevant to word finding in thalamic aphasia, the left inferior frontal gyrus (LIFG) has long been considered critical for language production. Some evidence indicates anterior to posterior processing gradient, with the anterior LIFG more involved in processing the semantic properties and the posterior LIFG in processing the phonological properties of words (Devlin, Matthews, & Rushworth, 2003; Klaus & Hartwigsen, 2019), which is consistent with our observation that generating category members shows a large volume of activity around the left inferior frontal sulcus (the superior boundary of pars triangularis, i.e., anterior LIFG) and generating rhyming words shows a large volume of activity around the left precentral sulcus, (the posterior boundary of left pars opercularis, i.e., posterior LIFG) (Crosson et al., 2003). Finally, it should be noted that the information which anterior LIFG receives as a part of its semantic role in word retrieval is already highly differentiated semantically (e.g., see Lambon Ralph, Jefferies, Patterson, & Rogers, 2017 for one viewpoint).

Even though processing moves from higher-order toward primary motor cortex, the laminar specifics of the semantic-lexical interface appear very similar to what we described above for sensory processing. However, some allowance must be made for the absent (agranular) or only rudimentary (dysgranular) layer 4 in some frontal cortices. Pars triangularis is considered eulaminate cortex (i.e., having all layers fully represented), but pars opercularis is considered dysgranular cortex (Schenker et al., 2008). Based on the schema of Barbas (2015), feedforward projections would emanate primarily from pars triangularis layers 2/3 and terminate on neurons in the rudimentary layer 4, upper layer 5, and perhaps deepest part of layer 3 in pars opercularis. Feedback would emerge primarily from layers 5/6 of pars opercularis and target neurons in pars triangularis layer 1 and upper layer 2. Bohsali et al. (2015) found that pars triangularis and pars opercularis connect with both the pulvinar and the ventral anterior thalamus, establishing the thalamic links for cortico-thalamo-cortical processing. Hence, the machinery to move from a semantic concept to selecting a word to represent it would look similar to the mechanisms that facilitate moving from a lexical to a semantic representation, as we discussed above for auditory-verbal comprehension. Specifically, in word finding (Fig. 6), semantic information from anterior LIFG is forwarded to posterior LIFG to select a phonological form for a word to represent the concept. As neural processing evolves toward word selection in posterior LIFG, a representation of the state of semantic processing from anterior LIFG is passed to posterior LIFG for comparison to emerging lexical possibilities, and error signals regarding those possibilities are fed back to anterior LIFG to facilitate any necessary refinement of the semantic representation.

We have not included basal ganglia connectivity through the ventral anterior thalamus as a contributor to the semantic-lexical interface even though basal ganglia loops are active during word finding (Crosson et al., 2003) and verbal working memory encoding (Moore et al., 2013). Briefly, the reasons are as follows: (1) Participation of such connectivity in a pre-SMA-basal ganglia loop happens during production based on lexical-phonological as well as semantic-lexical word-retrieval (Crosson et al., 2003). Hence, the contribution of this loop is not unique to semantic processing. (2) In basal ganglia lesion or disease, language deficits are subtle and more executive in nature (Copland, Chenery, & Murdoch, 2000; Crosson, Benjamin, & Levy, 2007; Isaacs, McMahon, Angwin, Crosson, & Copland, 2019). They are not characteristic of aphasia, which is the case in thalamic aphasia. (3) Acute left striato-capsular infarction, which usually occurs in large vessel (middle cerebral or even internal carotid artery) disease, does not cause aphasia unless accompanied by cortical under-perfusion (Hillis et al., 2002), which is related to inadequacy of anastomotic circulation which, when adequate, can back-fill the middle cerebral artery territory (Nadeau & Crosson, 1997; Weiller et al., 1993).

Finally, we should address the issue of long cortical connections. It is generally agreed that the semantic system is broadly distributed across brain areas, with some nodes showing category-specific processing preferences (e.g., Chao, Weisberg, & Martin, 2002; Chen, Lambon Ralph, & Rogers, 2017; Wierenga et al., 2009). The nature and extent of the distributed processes is a matter of debate (Chen et al., 2017; Reilly et al., 2014), though the idea that there is a dorsal stream for phonological processing and a ventral stream for semantic processing (Chen et al., 2017; Hickok & Poeppel, 2007) is gaining traction in the literature (McKinnon et al., 2018). For purposes of the current discussion, it is not necessary to resolve these controversies over how semantic processes are distributed; it is sufficient to know that anterior Broca’s area receives/exchanges highly processed semantic information from areas outside the frontal lobe, like the anterior temporal lobe and angular gyrus (Jakobsen et al., 2016; Lambon Ralph et al., 2017). This fact raises the question of whether there are higher-order cortico-thalamo-cortical relays for these longer connections. Because of its connectivity, the pulvinar is an excellent candidate for such a higher-order relay. The medial pulvinar receives fibers from and projects fibers to the inferior parietal lobe and ventral visual stream (Jones, 2007) as well as projecting to most frontal cortices (Goldman-Rakic & Porrino, 1985). Indeed, there is comingling of neurons that project to prefrontal cortex and those that project to parietal cortex in the medial pulvinar (Asanuma, Andersen, & Cowan, 1985). Using diffusion tractography, we have traced tracts between both anterior and posterior portions of Broca’s area and the pulvinar as well as the ventral anterior thalamus (Bohsali et al., 2015). Hence, based on what we know about higher order cortico-thalamo-cortical relays (e.g., Sherman & Guillery, 2006; Usrey & Sherman, 2019), we surmise with some confidence that such higher-order relays for longer connections (e.g., Broca’s area-pulvinar-parietal relays) probably exist. Such relays would allow pars triangularis (and/or pars orbitalis) to engage higher-level semantic processors outside the frontal lobe in refining lexical-semantic constructs during word selection and explain why thalamic aphasias and word-retrieval problems occur with pulvinar lesions (Ciemins, 1970; Crosson, Moberg, Boone, Rothi, & Raymer, 1997; Crosson et al., 1986; Kawahara et al., 1986; Mohr, Watters, & Duncan, 1975) and during electrical stimulation of the pulvinar (Ojemann, 1977; Ojemann, Fedio, & Van Buren, 1968).

## Why Lexical-Semantic Processing Is Affected by Thalamic Aphasia

Another key issue is why the most prominent deficits in thalamic aphasia lie at the semantic-lexical interface. In addition to clinical observations, this conclusion is based on extensive testing of two patients, one with an infarct in the left polar artery territory and the other with a left paramedian artery infarct, using the same 120 items on several different tasks,. Raymer et al. (1997) concluded that word-finding errors were lexical-semantic in origin. Specifically, neither patient had a more general semantic deficit as indicated by the fact that they could match words presented in the auditory modality to pictures with few or no errors. Further, the fact that neither patient had any errors in oral reading and very low numbers of errors in writing words to dictation indicates that they can match visual and auditory word forms with spoken and written word forms, respectively, suggesting that lexical systems generally were intact. However, both patients had substantially higher percentages of errors on word-retrieval tasks (oral picture naming, written picture naming, and naming to auditory definition) and errors were predominately semantic (including visual-semantic) in both cases. Further, the error rate was much higher for words with a low frequency of occurrence as opposed to a high frequency of occurrence in the English language. Hence, we can refine the above question to why interruption of feedforward processes specifically affects the semantic-lexical interface. Presumably, cortico-thalamo-cortical relays exist between other levels of language processing. For example, why wouldn’t a thalamic aphasia also affect the interface between lexical and phonemic processes in the word output stream? As noted above, phonemic errors do indeed occur in thalamic aphasias of ischemic origin (Perren, Clarke, & Bogousslavsky, 2005; Radanovic, Azambuja, Mansur, Porto, & Scaff, 2003), though they are less frequent than lexical-semantic errors across thalamic aphasia cases (Nadeau & Crosson, 1997).

The answer to this question relates to the complexity of the systems at the interface. Specifically, though estimates vary wildly, by one well-documented study, the average number of words known by English speaking college students is roughly 17,000 (D'Anna, Zechmeister, & Hall, 1991). The number of semantic concepts is harder to ascertain but is surely in the thousands. The number of phonemes in the English language is usually described as 40, though some would argue this is too many (Bizzocchi, 2017). Each of these systems has internal associations amongst its constituents: phonemes that occur frequently together (vs. those that do not) at the phonemic level, words that sound alike (e.g., rhyming words) or words that frequently co-occur at the lexical level, or concepts that have similarities in function, form, etc. for semantics. There are also relationships that connect items between systems: i.e., the phonemes that constitute a specific word and the meaning(s) a specific word may represent.

Put in the simplest terms, as one progresses toward execution in spoken language of a word to represent an associated concept, the interface between thousands of concepts and 17,000 words is much more massive and complex than the interface between 44 phonemes and 17,000 words and, therefore, more likely to spawn lexical-semantic errors than lexical-phonemic errors. As noted above, instances of phonemic and neologistic paraphasias are occasionally seen in thalamic aphasia. However, as suggested by the shear difference in the magnitude of the computational problem, lexical-semantic errors (including visual-semantic errors) are much more common than other errors (Crosson, 1992; Raymer et al., 1997). Finally, it should be noted that semantic errors (and acutely, semantic jargon) in thalamic aphasia are at the same time both an indication of what happens when stable thalamic representations of the state of semantic processing are no longer available to undergird semantic-lexical feedback processes and a glimpse into the first-pass at translating semantic concepts into lexical choices, before the cortico-thalamic recurrent circuitry has introduced feedback into word retrieval processes.

## Conclusions: From Inductive to Deductive Processes

This paper has focused on the role of higher-order thalamic relays (i.e., cortico-thalamo-cortical relays) in language processing. We have accomplished this goal primarily through inductive reasoning. Specifically, we have used both neuroanatomical and neurophysiological findings from animal model studies, as well as recent modifications of a long-standing conceptual framework (Hebb, 1949; Wang, 2008) to build a new model to explain the role of higher-order thalamic relays in language processing. The heuristic value of any neurocognitive model will depend on its ability to explain known phenomena and generate testable hypotheses. In the discussion in the last few paragraphs, we have dealt with why lexical-semantic errors in word-finding are more common than other word-finding errors. We also discussed how the role of specific thalamic nuclei in basal ganglia loops is relevant to word retrieval processes. Hence, it is appropriate to focus the following discussion on what additional phenomena the model can explain and what hypotheses it can generate.

In this regard, the model also should be able to explain why repetition usually is less impaired than other language functions in thalamic aphasia (Crosson, 1984, 1992). The answer is closely related to the above discussion of lexical-semantic errors. Essentially, the most direct route for word repetition is to access the spoken form for a word from its corresponding auditory pattern. If the word is known, then both forms are stored in their respective lexicons (Ellis & Young, 1988). Although the semantics related to these word forms may induce incidental activation during repetition, the semantic information is not essential to the process. In accessing the spoken word form for a word that is heard, the output is highly constrained by the input. In other words, the heard word form is so exclusively associated with its spoken version that less room for error exists as long as the form of the heard word is correctly decoded. It is worth noting that oral reading and writing words to dictation are very similar computational problems to repetition: they involve activating a spoken or written form from a visual or auditory word form, respectively, and can essentially bypass semantic activation. As noted above, Raymer et al. (1997) showed these two tasks produce few or no errors in cases of thalamic aphasia.

One potential weakness in the current model is the use of animal model data to infer mechanisms in a uniquely human function like language. For example, the specific information regarding at what cortical level cortico-cortical and cortico-thalamic pyramidal cells communicate was derived from rodent frontal and somatosensory cortices (Kawaguchi, 2017; Larsen & Callaway, 2006). Differences in connectivity patterns not only between different types of cortices, but also between species may exist. However, for purposes of the current discussion, it is sufficient to know that cortico-cortical pyramidal cells communicate with each other and cortico-thalamic pyramidal cells also communicate with each other. In other words, for the sake of completeness, we have covered evidence regarding axonal branching patterns for these kinds of neurons, but the necessary information is that these patterns of branching provide opportunities for communication between cortico-cortical neurons, as well as between cortico-thalamic neurons, and the ways in which these different classes of neurons influence each other is a clue to their function, as noted above.

In our description of the recurrent circuit at the semantic-lexical interface as a model for the functional role of higher-order thalamic relays in language, many readers probably have developed questions that could be tested in animal models. Yet, the purpose of the model is to explain phenomena related to language, a uniquely human function, and to generate further hypotheses within that realm. Hence, we will focus our closing remarks on future research directions regarding thalamic functions in language, which by definition must be tested in the human realm. Because of the limitation of methods available for use in humans, this endeavor can be challenging. For example, opportunities for doing recording from individual neurons are rare in humans, and opportunities for recording local field potentials are driven by the clinical need for such recordings. Nonetheless, hypotheses from the current model can be generated that are amenable to testing at a more macroscopic neural systems level using functional neuroimaging. Good examples of this kind of research do exist in the literature. For instance, Kraut, Hart and their colleagues (Kraut, Calhoun, Pitcock, Cusick, & Hart Jr., 2003; Kraut et al., 2002a, b), developed a semantic feature binding task that has provided insight into semantic functions when used with functional MRI. Briefly, they presented pairs of semantic features either through pictures or words, and participants press one button if the features can be combined to make an object (e.g., desert + humps make camel) and another button if the features cannot be combined in this fashion (e.g., bullets + milk do not form an object). For features that do combine to make an object, the dorsal, posterior thalamus is active, but it is not active for features that cannot be combined to make an object. Further, activity is mostly limited to the left thalamus when the stimulus pairs making an object are both words. These findings are consistent with studies indicating that lesions of the posterior thalamus cause thalamic aphasias with the usual pattern of semantic paraphasias (Crosson, 1984, 1992; Crosson et al., 1986).

Another issue requiring further attention is why patients with lesions confined to the thalamus recover relatively rapidly. One possibility, specific to polar artery infarcts, is that the lesion is near a critical pathway between frontal cortices and the pulvinar. Bohsali et al. (2015) traced this pathway, and it is, indeed, very close to the location a polar artery lesion would occupy (Nishio et al., 2014). In the acute phase, the edema and related processes from polar artery lesions could disrupt transmission of information down this critical pathway, but as this perilesional disruption subsides during the sub-acute phase, language processing rapidly recovers, albeit incompletely. A second possibility is redundancy of connections providing multiple pathways for cortico-thalamo-cortical transmission. For example, Goldman-Rakic and Porrino (1985) noted three nuclei to which all frontal regions project: the ventral anterior nucleus, the dorsal medial nucleus, and the medial pulvinar. If the primary cortico-thalamo-cortical pathway is disrupted, a subordinate circuit with similar connectivity could take over. One way to distinguish these possibilities might be to do serial resting-state fMRI imaging comparing polar artery cases to normal controls. If the first possibility is the correct answer, then we should see reduced connectivity between the pulvinar and the frontal lobes in the acute phase. But, as the behavior improves, this connectivity should return to more normal levels. However, if the second possibility is the correct answer, then frontal functional connectivity with the remaining portions of the ventral anterior nucleus should be low in both the acute and subacute phases. But, as language processes recover, the connectivity between another thalamic location (probably the dorsal medial nucleus or the pulvinar) and the frontal lobe should increase. Such an experiment would require careful monitoring for left polar lesions in a relatively large thalamic stroke large sample.

In closing, this neurocognitive model can provide a framework to develop hypotheses for future research, including lesion studies. A few conceptually driven studies exist in the literature (e.g., Mennemeier et al., 1997; Raymer et al., 1997), but this is an underexploited area of research. What is missing is longitudinal data addressing the evolution of lexical-semantic deficits in thalamic aphasia and studies of language function in larger numbers of patients with thalamic aphasia. Also, as noted above, longitudinal studies of functional connectivity in thalamic lesions would assist us in understanding what mechanisms account for the rapid recoveries usually seen in thalamic aphasia. Finally, the current conceptual framework is amenable to mathematical modeling, which could yield further insights regarding the properties necessary for this system to function efficiently and how it breaks down after dominant thalamic lesion.
